# Institutional delivery service utilization in Munisa Woreda, South East Ethiopia: a community based cross-sectional study

**DOI:** 10.1186/1471-2393-12-105

**Published:** 2012-10-08

**Authors:** Abdella Amano, Abebaw Gebeyehu, Zelalem Birhanu

**Affiliations:** 1 Department of Midwifery, College of Medicine and Health Sciences, the University of Gondar, Gondar, Ethiopia; 2 Department of Reproductive Health, Institute of Public health, the University of Gondar, Gondar, Ethiopia

## Abstract

**Background:**

Reducing maternal morbidity and mortality is a global priority which is particularly relevant to developing countries like Ethiopia. One of the key strategies for reducing maternal morbidity and mortality is increasing institutional delivery service utilization of mothers under the care of skilled birth attendants. The aim of this study was to determine the level of institutional delivery service utilization and associated factors.

**Methods:**

A community-based cross-sectional survey was conducted from April 1–20, 2011, among mothers who gave birth 12 months before the study began in Munesa Woreda, Arsi Zone, Oromia Region, Southeast Ethiopia. A stratified cluster sampling was used to select a sample of 855 participants.

**Results:**

Out of all deliveries, only 12.3% took place at health facilities. Women who were urban residents (AOR = 2.27, 95%CI: 1.17, 4.40), women of age at interview less than 20 years (AOR = 6.06, 95%CI: 1.54, 23.78), women with first pregnancy (AOR = 2.41, 95%CI: 1.17, 4.97) and, women who had ANC visit during the last pregnancy (AOR = 4.18, 95%CI: 2.54, 6.89) were more likely to deliver at health institutions. Secondary and above level of mother`s and husband`s education had also a significant effect on health institution delivery with AOR = 4.31 (95%CI: 1.62, 11.46) and AOR = 2.77 (95%CI: 1.07, 7.19) respectively.

**Conclusion:**

Institutional delivery service utilization was found to be low in the study area. Secondary and above level of mother`s and husband`s education, urban residence and ANC visit were amongst the main factors that had an influence on health institution delivery. Increasing the awareness of mothers and their partners about the benefits of institutional delivery services are recommended.

## Background

Maternal mortality remains unacceptably high across much of the developing world. An estimated 358, 000 maternal deaths occurred worldwide in 2008. Sub-Saharan Africa (SSA) and South Asia accounted for 87% of the global maternal deaths [1]. In sub-Saharan Africa, a woman’s risk of dying from treatable or preventable complications of pregnancy and childbirth over the course of her lifetime is 1 in 22, compared to 1 in 7,300 in the developed regions [2]. In Ethiopia, maternal mortality and morbidity levels are among the highest in the world. The Maternal Mortality Ratio (MMR) in 2005 was 673 per 100,000 live births [3].

Around 80% of maternal deaths worldwide are brought about by such direct causes as hemorrhage, infection, obstructed labor, unsafe abortion and high blood pressure. Severe bleeding which usually occurs after the mother has given birth is the single most feared complication claiming the lives of most mothers [4].

Globally, the annual percentage decline in MMR between 1990 and 2008 was only 2.3%. Most SSA countries are not on track to meet the Millennium Development Goal (MDG) targets pertaining to MMR because recent estimates suggest that the average annual rate of reduction in MMR for these countries is 1.7% [1].

Three-quarters of maternal deaths occur during childbirth and the post-partum period. Most of the maternal deaths will be avoidable if women have access to vital health care before, during, and after childbirth [2]. One of the indicators of meeting MDG5 is the proportion of women who deliver with the assistance of skilled birth attendants. In almost all countries, where health professionals attend more than 80% of deliveries, maternal mortality ratios are below 200 per 100,000 live births [5]. A skilled birth attendant at delivery is critical to reducing maternal deaths. In 2006, nearly 61% of the births in the developing world were assisted by skilled birth attendants. However, coverage remains low in Southern Asia (40%) and SSA (47%) – the two regions with the greatest number of maternal deaths [2].

The proportion of births attended by skilled attendants in Ethiopia is very much lower than that of countries in SSA. Even for women who have access to the services, the proportion of births occurring at health facilities is very low. Nationally, only 6% of the births took place at health institutions according to the 2005 DHS (Demography and Health Survey), and there was no significant difference in proportion of institutional delivery service utilization between DHS 2000 and 2005. In Oromia Region, institutional delivery service utilization which is about 4.8%, is lower than that of the national level [3]. Therefore, assessing the factors affecting institutional delivery service utilization in the study area is very important to improve maternity services and thereby reduce maternal and infant deaths.

## Methods

A community-based cross-sectional study was conducted in Munesa Woreda from April 1–20, 2011. The Woreda is found in Arsi Zone, Oromia Regional State. Kersa, the capital of the Woreda is located 232km from Addis Ababa, the capital of Ethiopia and 57 km from the capital of Arsi Zone, Asella. Organized into 32 rural and 3 urban kebeles, the woreda has 2 health centers, 4 medium clinics, and 32 health posts. According to the 2007 census, it has a total population of 166,414 [6].

The participants of the study were mothers who delivered 12 months before the study began. Women who delivered in those months, especially those who reported to have delivered after 28 weeks of gestation, were included in the study regardless of the outcomes of the births. The stratified cluster sampling technique was used to select the study units. That is, by taking kebeles as clusters, one of the three urban and eight of the 32 rural kebeles were selected by simple random sampling technique, making it possible to accommodate all eligible mothers in the clusters.

The sample size was determined by using a single population proportion formula which took the following assumptions in to consideration: magnitude of institutional delivery service utilization 7%, [7] (**p** = 0.07); 5% level of significance (α = 0.05); 2.5% marginal error (**d** = 0.025). The final sample size was adjusted by using the design effect of 2 and 5% non-response rate. Thus, the sample size turned out to be 855.

Data was collected through face to face interviews using a structured and a pre-tested questionnaire. Ten health science students were collected data, and, the house to house survey was carried out to get eligible mothers. Three nurses from Kersa Health Center were assigned to supervise the data collection process after they were trained, together with the data collectors.

Data entry was done by using EPI Info 2002 and exported to SPSS version 16.0 software package for analysis. The data were analyzed using logistic regression to determine the effect of various factors on the outcome variable and to control confounding effects. The results were presented in the form of tables, figures and texts using frequencies and summary statistics such as mean, standard deviation, and percentage to describe the study population in relation to relevant variables(age, residence, ethnicity, religion, marital status, educational status, occupational status, distance from health center, parity, family size, ANC, and place of delivery). Valid categories were employed for the variables used in the context from EDHS and other published literature. The strength of association between independent and dependent variables was assessed using the odds ratio with 95% confidence interval.

The expression “skilled attendant” in this study means a delivery attended by skilled health providers in the community or at a health institution. But in Ethiopia, all deliveries which occur at health institutions are attended by skilled birth attendants while such skilled care is rare in the community.

Ethical clearance was obtained from the School of Public Health, the University of Gondar. A formal letter request of cooperation was written to Arsi Zone Health Department and Munesa Woreda Health Office. Verbal consent was obtained from each study participant.

## Results

### Socio-demographic characteristics

A total of 855 mothers who gave birth in the last 12 months were interviewed; out of these 739 (86.4%) were rural dwellers. The mean age of the respondents was 27.22 years with a standard deviation (SD) of 5.87 years. The majority (96.7%) of the mothers were married. Five hundred twenty seven (61.6%) of the mothers and 324 (37.9) of their husbands were unable to read and write. Seven hundred twenty-three (84.6%) of the female respondents were housewives, while 726 (84.9%) of the husbands were farmers.

Six hundred twenty nine (73.6%) of the respondents had either radio or Tv set in their houses. To reach the nearest health center, 719 (84.1%) of the participants had to walk for 30 minutes on average (Table 1).

**Table 1:** Socio-demographic characteristics of the study participants, Munesa Woreda, Arsi Zone, southeast Ethiopia, April 2011

**Variables**	**Frequency (%)**
**Age of the mothers at interview**	
15-19	50 (5.9)
20-24	224 (26.2)
25-29	268 (31.3)
30-34	178 (20.8)
35+	135 (15.8)
**Marital status**	
Married	838 (98)
Divorced/separated	17 (2.0)
**Distance from health facility**	
≤ 30 minute	136 (15.9)
>30 minute	719 (84.1)
**Religion**	
Orthodox	442 (51.7)
Muslim	371 (43.4)
Protestant	42 (4.9)
**Ethnicity**	
Oromo	834 (97.5)
Amhara	17 (2.0)
Gurage	4 (0.5)
**Educational status of the mother**	
Unable to read and write	527 (61.6)
Primary education	306 (35.8)
Secondary and post secondary	22 (2.6)
**Occupational status of the mother**	
House wife	746 (87.3)
Government employee	12 (1.4)
Farmer	97 (11.3)
**Occupational status of the husband**	
Farmer	726 (84.9)
Government employee	30 (3.5)
Daily laborer/Merchants	99 (11.6)
**Educational status of the husband**	
Unable to read and write	324 (37.9)
Primary education	487 (57.0)
Secondary and post secondary	44 (5.1)
**Perceived economical status**	
Low	220 (25.7)
Medium	422 (49.4)
High	213 (24.9)
**Family size**	
≤5	412 (48.2%)
>5	443 (51.8)

### Obstetric characteristics

**Figure 1:**
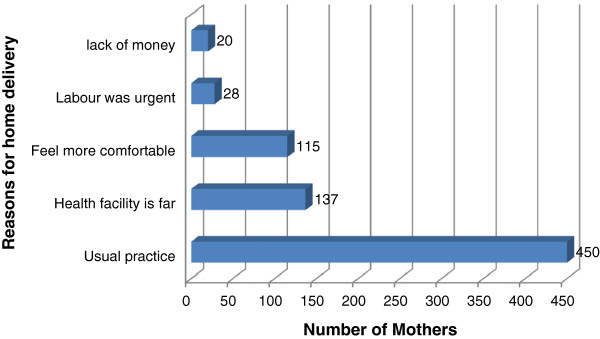
Reasons for Home delivery among mothers who gave birth in the last 12 months in Munesa woreda, Arsi zone, South East Ethiopia, April, 2011.

Six hundred seventeen (72.2%) of the mothers had their first pregnancy before the age of 20 years, and the minimum and maximum ages at first pregnancy were 14 and 37 years with a mean and SD of 18.51 and 2.31 years, respectively. During the last pregnancy, 297 (34.7%) of the mothers had at least one ANC (Antenatal Care) visit. Out of the total respondents, only 105 (12.3%) gave birth at health facilities (hospitals and health centers), and the vast majority (87.7%) delivered at home. Among the mothers who delivered at home, 392 (52.2%) were assisted by their families or relatives; 23 (3.1%) delivered without any assistance. Of those who went to health facilities, 66 (62.9%) delivered at health centers, 27 (25.6%) at hospital, and the rest (11.5%) at private clinics (Table 2). Mothers gave a variety of reasons for delivering at home. For example, 450 (52.6%) said that delivering at home was the norm or the usual practice (Figure 1).

**Table 2:** Obstetric characteristics of respondents, Munesa woreda, Arsi Zone, southeast Ethiopia, April 2011

**Variables**	**Frequency**	**Percent**
**Age at first pregnancy(years)**		
<20	617	72.2
≥20	238	27.8
**Parity**		
One	139	16.3
Two-four	390	45.6
Five and above	326	38.1
**Ever had abortion**		
Yes	62	7.3
No	793	92.7
**Ever had still birth**		
Yes	77	9.0
No	778	91.0
**ANC visit during last pregnancy**		
Yes	297	34.7
No	558	65.3
**Number of ANC visits**		
One	12	4.0
Two to Three	123	41.4
Four and above	162	54.6
**Place of last 12 month delivery**		
Home	750	87.7
Health facility	105	12.3
**Assistance during home delivery**		
No one	23	3.1
Trained TBAs	47	6.3
Untrained TBAs	288	38.4
Family or relatives	392	52.2

### Factors associated with health institution delivery service utilization

As shown in the bivariate models, institutional service delivery utilization was significantly associated with the age, residence, occupational and educational status of the mothers, and the occupational and educational status of the husbands as well as with distance from the nearby health centers, family size, parity, and ANC visit during the last pregnancy.

On the basis the variables found to be significant in the bivariate analysis, maternal age at interview, residence, educational status of couples, ANC visit during the last pregnancy and parity were significantly associated with institutional delivery service utilization in multiple logistic regression analysis, too.

Mothers less than 20 years of age during the interview were about 6 times (AOR = 6.06, 95%CI: 1.54, 23.78) more likely to deliver at health institutions than mothers more than 35 and above. Urban mothers were about 2.3 times more likely to deliver at health institutions than rural mothers (AOR = 2.27, 95%CI: 1.17, 4.40). Mothers with secondary education and above were 4.3 times more likely to deliver at health facilities as compared to those who were not able to read and write (AOR = 4.31, 95%CI:1.62,11.46).

Regarding the educational status of husbands, mothers whose husband attended secondary school and above were 2.8 times (AOR = 2.77, 95%CI:1.07, 7.19) more likely to deliver at health institutions as compared to mothers whose husbands were unable to read and write.

ANC visit during the last pregnancy was also found to be a strong predictor of institutional delivery service utilization. Mothers who visited health facilities for ANC during pregnancy were 4.2 times (AOR = 4.18, 95%CI: 2.54, 6.89) more likely to deliver at health institutions than those who did not visit ANC during the last pregnancy. Parity was also another important factor which was associated with the place of delivery. Mothers who were delivering their first babies were 2.4 times more likely to utilize health institutions as compared to those who had five and more deliveries (AOR = 2.41, 95%CI: 1.17, 4.97) (Table 3).

**Table 3:** Bivariate and multivariate analysis of factors associated with institutional delivery service utilization in Munesa woreda, April 2011

**Variables**	**Institutional delivery**		**COR (95%CI)**	**AOR (95%CI)**
	**Yes**	**No**		
**Place of residence**				
Urban	26	90	2.41 (1.47,3.96	2.27 (1.17,4.40)
Rural	79	660	1	1
**Distance from health institution**				*
≤30minute	27	109	2.04 (1.26,3.30)	
>30minute	78	641	1	
**Age of the mother**				
<20	15	35	14.04 (4.38,44.9)	6.06 (1.54,23.78)
20-34	86	584	4.80 (1.74,13.39)	4.02 (1.34,12.06)
35+	4	131	1	1
**Possession of Radio or TV**				*
Yes	79	550	1.10 (0.69,1.77)	
No	26	200	1	
**Occupational status of the mother**				*
House wife	88	658	1	
Government employee	4	8	3.74 (1.10,12.67)	
Farmer	13	84	1.16 (0.62,2.16)	
**Family size**				*
≤5	72	340	2.63 (1.70,4.07)	
>5	33	410	1	
**Educational status of the mothers**				
Unable to read and write	50	477	1	1
Primary education	47	259	1.73 (1.13,2.65)	1.44 (0.92,2.24)
Secondary and post secondary	8	14	5.45 (2.18,13.63)	4.31 (1.62,11.46)
**Occupational status of the husbands**				*
Farmer	74	652	1	
Government employee	7	23	2.68 (1.11,6.64)	
Daily laborer/Merchants	24	75	2.82 (1.68,4.74)	
**Educational status of the husbands**				
Unable to read and write	30	294	1	1
Primary education	59	428	1.35 (0.85,2.15)	0.61 (0.36,1.03)
Secondary and post secondary	16	28	5.60 (2.73,11.50)	2.77 (1.07,7.19)
**Parity**				
1	35	104	4.65 (2.61,8.29)	2.41 (1.17,4.79)
2-4	48	342	1.94 (1.14,3.29)	0.99 (0.55,1.80)
5+	22	304	1	1
**ANC visit during last pregnancy**				
Yes	74	223	5.64 (3.61,8.83)	4.18 (2.54,6.89)
No	31	527	1	1

* Not significant in back ward stepwise logistic regression.

## Discussion

The results of the study revealed that the proportion of women who delivered at health facilities was 12.3% in the Woreda, and that the vast majority of mothers (87.7%) gave birth at home. This finding was low when compared with the national MDG reports of 2010 which showed that the percentage of deliveries attended by skilled health personnel was 25 [8]. The current utilization was higher than the national and the Oromia Region EDHS result of 2005 which were 6% and 4.8%, respectively [3]. This might be due to the time gap i.e. since 2005 there might have been improvements in accessing and utilizing of the service. The result was in line with that of a study done in North Gondar Zone in 2002, which was 13.5% [9]. However, it was lower than those of studies conducted in Enugu, Nigeria and southern Tanzania where the proportion of women who gave birth at health facilities was 47.1% and 46.7%, respectively. The difference could be explained by the fact that mothers in these countries had better educational status and better ANC service utilization [10,11].

Residence of the respondents was significantly associated with institutional delivery service utilization. Mothers who lived in urban kebeles were about 2.3 times more likely to deliver in health institutions as compared to those who lived in rural kebeles. The finding was consistent with EDHS 2000 and that of a study done in North Gondar Zone [9,12]. The study in Nigeria also showed that urban/rural differences had significant associations with the place of delivery [10]. This might be because in urban areas, the proportion of educated mothers was higher; they get maternal and other health services nearby and had better access to information than rural mothers.

Maternal age was also one of the predictors of institutional delivery service utilization. Mothers with less than 20 years of age at the time of the interview were about 6 times more likely to give birth in health institutions than mothers aged 35 years and above. This finding was in line with studies done in North Gondar Zone, Kenya, and Afghanistan which showed that younger women were more likely to utilize institutional delivery service as compared to older ones [9,13,14]. The possible explanation for this could be that younger women were more likely to be more literate than older women, and that older women tended to consider giving birth at home not so risky as it has been their usual experience. Furthermore, older women might belong to a more traditional cohort and thus be less likely to use modern facilities as compared to younger women.

The results of this study showed that, institutional delivery service was significantly influenced by the level of education. Women with higher level of education (secondary and above) were about 4.3 times more likely to deliver at health facilities than those who were unable to read and write. This finding is similar with those of studies conducted in different parts of Ethiopia [9,12], Bangladesh, Nigeria, and Afghanistan [10,14-16]. These might be due to the fact that educated women had better awareness about the benefits of preventive health care and health services. They might also have higher receptivity to new health-related information. Familiarity with modern medical culture, egalitarian relationship and better communication with husbands, more decision-making power, increased self-worth and self-confidence were also the characteristics of urban women which might have contributions to better utilization of health facility delivery than rural women.

Husbands’ educational level was also one of the factors that predicted health institution delivery. Women whose husbands had secondary and post-secondary education were about 2.8 times more likely to deliver in health facilities as compared to those whose husbands were unable to read and write. Our finding was in line with that of a study done in Enugu-northeastern Nigeria and Zaria, northern Nigeria [10,16]. The possible explanation for this might be that educated husbands might be more open toward modern medicine and aware of the benefits of giving birth at health facilities. They might also put fewer constraints on their wives' mobility and decision-making, thus facilitating care-seeking.

ANC services can provide opportunities for health workers to promote a specific place of delivery or give women information on the status of their pregnancy which in turn alerts them to decide where to deliver. This study showed that mothers who had ANC visits during the last pregnancy were about four times more likely to deliver at health facilities compared to those who did not have any visits. The result was consistent with other studies done in North Gondar Zone, India, and Mali, which revealed that mothers who attended ANC follow up were more likely to deliver at health facilities than those who did not [9,17,18].

As a cross-sectional study requires respondents to remember information retrospectively, recall and interviewer bias are the potential limitations of this study. However, numerous scientific procedures have been employed to minimize the possible effects. To reduce the recall bias, for instance, only women who gave birth in the last one year were selected. In addition, procedures such as supervision, pretest of data collection tool, and adequate training of data collectors and supervisors were utilized. Wide confidence intervals observed in some of the variables are due to the calculation of the intervals by the sample size calculated for the prevalence.

## Conclusions

This study revealed that the proportion of women who gave birth at health facilities in the woreda was low. Urban residence, age below 20 years at the time of interview, parity, ANC visit during the last pregnancy, secondary and post-secondary levels of education of mothers and husbands were factors significantly associated with institutional delivery service utilization. Increasing the awareness of mothers and their partners about the benefits of institutional delivery services are recommended.

## Competing interests

The authors declare that they have no competing interests.

## Authors’ contributions

AA wrote the proposal, participated in data collection, analyzed the data and drafted the paper. AG and ZB approved the proposal with some revisions, participated in data analysis and revised subsequent drafts of the paper. All authors read and approved the final manuscript.

## Pre-publication history

The pre-publication history for this paper can be accessed here:

http://www.biomedcentral.com/1471-2393/12/105/prepub

## References

[B1] WHO UNICEF UNFPA and the World Bank Trends in Maternal Mortality: 1990 to 2008 2010 Geneva: WHO, UNICEF, UNFPA and the World Bank

[B2] United Nations Millennium Development Goals Report 2008 New York: United Nations

[B3] Central Statistical Agency [Ethiopia] and ORC Macro Ethiopia Demographic and Health Survey 2005 2005 Addis Ababa: Central Statistical Agency and ORC Macro

[B4] Hogan MC Foreman KJ Naghavi M Maternal Mortality for 181 countries, 1980–2008: a systematic analysis of progress towards MDG5 Lancet 2010 375 9726 1609 1623 10.1016/S0140-6736(10)60518-1 20382417

[B5] WHO Maternal Mortality in 2005 2007 Geneva: WHO, UNICEF and UNFPA

[B6] FDRE, Census Commission Summary and statistical report of the 2007 population and housing census 2008 Addis Ababa: FDRE (Federal democratic republic of Ethiopia) census commission

[B7] FMOH, UNICEF, UNFPA, WHO and AMDD National Baseline Assessment for Emergency Obstetric & Newborn Care in Ethiopia 2008 Addis Ababa: The Ethiopian federal Ministry of health

[B8] Ethiopia MDG report Trends and Prospects for meeting MDG by 2015 2010 Addis Ababa: Federal Ministry of Health

[B9] Nigussie M Haile Mariam D Mitike G Assessment of safe delivery service utilization among women of childbearing age in north Gondar Zone, North West Ethiopia Ethiop J Health Dev 2004 18 3 145 152

[B10] Onah HE Ikeako LC Iloabachie GC Factors associated with the use of maternity services in Enugu, south-eastern Nigeria Soc Sci Med 2006 63 7 1870 1878 10.1016/j.socscimed.2006.04.019 16766107

[B11] Mpembeni RNM Killewo JZ Leshabari MT Use pattern of maternal health services and determinants of skilled care during delivery in Southern Tanzania: implications for achievement of MDG-5 targets BMC Pregnancy and Childbirth 2007 7 29 7 18053268 10.1186/1471-2393-7-29 PMC2222241

[B12] Mekonnen Y Mekonnen A Factors Influencing the Use of Maternal Healthcare Services in Ethiopia J Health Popul Nutr 2003 21 4 374 382 15038593

[B13] Van E Hanneke MB Frank O Use of antenatal services and delivery care among women in rural Western Kenya: a community based survey Reproductive Health 2006 3 2 3 16597344 10.1186/1742-4755-3-2 PMC1459114

[B14] Mayhew M Hansen M Peters D Determinants of Skilled Birth Attendant Utilization in Afghanistan: a Cross-Sectional Study Am J Public Health 2008 98 10 1849 1856 10.2105/AJPH.2007.123471 18703445 PMC2636465

[B15] Kamal Mostafa SM Factors affecting utilization of maternity care services among married adolescents in Bangladesh Asian Population Studies 2009 5 2 153 170 10.1080/17441730902992075

[B16] Sheshu AU Gwarzo Indris SH Determinants of place of delivery among semi-urban women in Northern Nigeria Ann Afr Med 2006 5 2 68 72

[B17] Gage AJ Barriers to the utilization of maternal health care in rural Mali Soc Sci Med 2007 65 8 1666 1682 10.1016/j.socscimed.2007.06.001 17643685

[B18] Sugathan KS Mishra V Retherford RD Promoting Institutional Deliveries in rural India: The Role of Antenatal-Care Services. National Family Health Survey Subject Reports Number 20 2001 India: International Institute for Population Sciences Mumbai

